# Inguinal Mesh Hernioplasties: A Rural Private Clinic Experience in South Eastern Nigeria

**DOI:** 10.5539/gjhs.v5n4p176

**Published:** 2013-05-13

**Authors:** Michael Enyinnah, Paul Owajionyi Dienye, Patrick Njoku

**Affiliations:** 1 Department of Surgery, Federal Medical Centre, Umuahia, Nigeria; 2 Department of Family Medicine, University of Port Harcourt Teaching Hospital, Port Harcourt, Nigeria; 3 Department of Family Medicine, Federal Medical Centre, Umuahia, Nigeria

**Keywords:** inguinal mesh hernioplasties, rural practice, Nigeria

## Abstract

**Objective:**

The objective of this paper is to review hernioplasties done for inguinal hernias in a rural private hospital, bringing out the socio-demographic and clinical pattern and to sensitize surgeons and family physicians in our environment about the possibility of making hernioplasty a standard of care for inguinal hernias.

**Method:**

The records of seventy seven patients operated in a rural private hospital were reviewed. Socio-demographic data, operative techniques and post-operative outcomes were documented. The results were compared with relevant findings in the literature.

**Results:**

Eighty one Lichtenstein procedures were done, of which four were bilateral. Polypropylene mesh was used in all cases. A total of three patients (3.9%) had early post-operative complications. The complications were scrotal haematoma, haematoma complicated by wound sepsis and wound sepsis only. All the complications were successfully managed. There was no case of mesh removal or mortality.

**Conclusion:**

Early post-operative results suggest that mesh hernioplasty is possible in rural communities of West Africa, given the availability of mesh, basic medical infrastructure and relevant skilled manpower.

## 1. Introduction

Repair of inguinal hernias evolved over the years from different types of suture repairs to the current practice of prosthetic repair using different kinds of mesh. The main short-coming of suture or non-mesh repair is the unavoidable tension created along the suture lines and in the surrounding tissues incorporated in the repair, which in turn is the principal cause of recurrence (Malangoni & Rosen, 2008).

Inguinal mesh hernioplasty involves strengthening the posterior inguinal wall using mesh, and is tension-free (Watkin & Robertson, 2006; Khan, Naeem, Bargash, Sadiq, & Hamid, 2008). Not all cases of mesh repair of the posterior wall of the inguinal canal constitute Lichtenstein repair. Lichtenstein repair is just one type of mesh repair. It is an open procedure in which polypropylene mesh is sutured to the pubic tubercle, conjoint tendon/muscle and inguinal ligament thereby supporting the inguinal muscular layers (Lichtenstein, Shulman, Amid, & Montllor, 1989). There are other types of mesh repair of the posterior wall of inguinal ligament like laparoscopic repairs (totally extraperitoneal repair-TEP, transabdominal preperitoneal repair-TAPP), mesh plug, prosthetic hernia system. These are all forms of mesh repair but not Lichtenstein procedure.

It is believed that mesh allows and supports good fibroblast proliferation which in turn strengthens the weak posterior wall of the inguinal canal (Sriram, 2009). Mesh for hernia repair was introduced in the 1950's while in 1986 Lichtenstein described its use in inguinal hernias (Watkin & Robertson, 2006; Khan et al., 2008; Farquharson & Moran, 2005). Mesh hernioplasty is becoming the prime treatment for inguinal hernia (Malangoni & Rosen, 2008). Compared to endoscopic methods, it makes inguinal hernia repair faster, easier and reduces recurrence rates drastically (Watkin & Robertson, 2006). Other advantages of mesh inguinal repair include less postoperative pain and early return to work (Farquharson & Moran, 2005; Shi, Su, Li, Liu, & Jing, 2010). Under local anaesthesia, it can be performed as a rapid outpatient procedure with cost saving (van Veen et al., 2008). In the developed countries, the choice is between open and laparoscopic procedures.

It is important that in Lichenstein repair the mesh is fixed to the pubic tubercle, conjoint tendon and inguinal ligament using interrupted non-absorbable sutures. There is the need for absolute homeostasis and prevention of infection in inguinal hernioplasty (Sriram, 2009). The post-operative and peri-operative complications associated with Lichtenstein mesh inguinal hernia repair are uncommon and include seroma and haematoma formation, wound sepsis, testicular atrophy, foreign body reaction, pain, fistula formation, migration, shrinkage and recurrence (Khan et al., 2008). A major limitation for inguinal hernioplasty is the cost of its procurement especially in resource poor countries like Nigeria and where there is gross contamination as is the case in strangulated hernias with gangrenous bowel (Watkin & Robertson, 2006; Farquharson & Moran, 2005).

A review of the literature had shown that very few studies (Ngowe et al., 2005; Arowolo et al., 2011) had been conducted in this part of the world despite the increasing inguinal hernia repairs observed mostly in the rural areas where agriculture is the principal occupation of the people.

This study was intended to highlight the experience with mesh inguinal hernioplasty in a rural setting where there is poor infrastructure and poor agricultural techniques which are known to cause and aggravate adverse health conditions. This study will also underscore the need for general surgeons and family physicians to help reduce recurrence of inguinal hernias and the morbidity associated with it by adopting this technique of hernia repair.

## 2. Materials and Method

This study was conducted at Crisrhod clinic, a rural private hospital located at Ukpakiri village, about five kilometres from Aba, along Aba -Ikot Ekpene road in Abia State, South Eastern Nigeria. The village lacks basic amenities such as pipe-borne water and well equipped health facilities. The major occupation of the people was farming. The clinic had a full staff of efficient primary-care medical team including a general surgeon. The service was general-practice, primary-care oriented, and therefore a wide range of patients (surgical, medical, gynecological and pediatric) were seen. A lot of patients with inguinal hernias were seen in this clinic. This was due to the fact that the patients were mainly farmers and were predisposed to this disease condition. Lichtenstein repair was the preferred method of hernia repair in the hospital due to the availability of mesh materials which were donated by an American surgeon who is a friend of the principal author. Spinal anesthesia (0.5% bupivacaine) was used in all cases and this was given by the surgeon. This was because from the surgeon's previous experience local anaesthesia, which is widely favoured, gave inconsistent results as regards patients’ co-operation and relaxation during surgery. All the patients received pre-operative intravenous antibiotics consisting of intravenous ampiclox one gramme and 80 milligramme of gentamicin. In some cases, this was continued for varying periods post-operatively depending on the estimated risk of infection.

Post-operatively, patients were given intramuscular pentazocine for analgesia. Intravenous fluid was stopped on full recovery from anaesthesia and patients commenced on oral intakes.

Patients were offered early discharge but were allowed to stay till non-absorbable skin sutures were removed, if they so desired. Generally, those that felt well early and asked for early discharge were granted. Initial follow-up was usually within two weeks but those that left the hospital early were seen for skin suture removal between seven and ten days post surgery.

The medical records of all the inguinal hernioplasty patients in the clinic over two years (May 2007 to April 2009) were reviewed and information on patients were extracted from them and transferred to a data sheet. The data collected included socio-demographic characteristics, past history of hernia, family history of hernia, inguinal side affected, duration of symptoms and management. The data was analyzed using the SPSS version 15 package and presented in tables. P-value of 0.05 was used to determine the statistical significance.

## 3. Results

**Figure 1:**
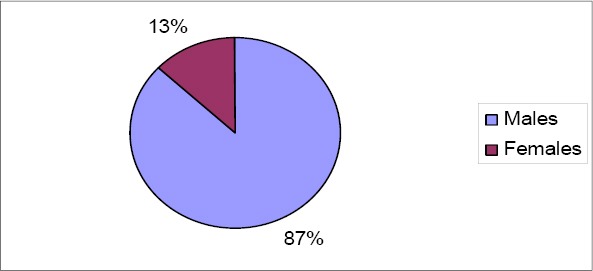
Sex distribution of patients

A total of eighty one mesh inguinal hernioplasties were done on seventy seven patients as there were four bilateral procedures. The hospital records of seventy-seven patients were therefore reviewed. Majority of the patients were males (87.0%). The male to female ratio was 6.7 to 1 (Figure 1).

The ages of the patients ranged from 20 years to 80 years with a mean of 46.8 ± 15.7 years. The mean age of the males was 46.2 ± 15.6 years, while that of females was 50.8 ± 16.8 years. There was no significant statistical difference in the mean ages of both sexes (t = 0.859, p-value = 0.804) (Table 1). The largest proportion of the patients were farmers (25.6%). This was followed by traders (24.4%). Engineers, tailors and clergy men constituted 3.8% each (Table 2).

**Table 1:** Age distribution of patients

Age (in years)	Male (%)	Female (%)	Total (%)
≤29	12(17.9)	1(10.0)	13(16.8)
30-39	8(11.9)	0(0.0)	8(10.4)
40-49	21(31.3)	3(30.0)	24(31.2)
50-59	10(14.9)	3(30.0)	13(16.8)
60-69	10(14.9)	1(10.0)	11(14.3)
≥70	6(9.0)	2(20.0)	8(10.4)

**Table 2:** Distribution of occupation

Occupation	Male (%)	Female (%)	Total (%)
Farmer	15(22.4)	5(50.0)	20(25.6)
Trader	16(23.9)	3(30.0)	19(24.4)
Driver	8(11.9)	0(0.0)	8(10.3)
C/Servant	6(9.0)	0(0.0)	6(7.7)
Artisan	5(7.5)	0(0.0)	5(6.8)
Student	3(4.5)	1(10.0)	4(5.1)
Engineer	3(4.5)	0(0.0)	3(3.8)
Tailor	3(4.5)	0(0.0)	3(3.8)
Clergy	2(3.0)	1(10.0)	3(3.8)
Others	6(9.0)	0(0.0)	6(7.7)

Others include welders, contractors, herbalists, laboratory technicians and security personnel.

The largest proportion of the patients (27.3%) had their symptoms lasting more than two years before presentation. However only a small proportion of patients (16.8%) did not have their symptom duration recorded (Table 3).

**Table 3:** Duration of symptoms at presentation

Duration of symptoms(Yrs)	Males (%)	Females (%)	Total (%)
<1	13(19.4)	2(20.0)	15(19.5)
1	12(17.9)	1(10.0)	13(16.8)
2	13(19.4)	2(20.0)	15(19.5)
>2	15(22.4)	6(60.0)	21(27.3)
Not recorded	13(19.4)	0(00)	13(16.8)

There was greater affectation of the right groin (54.5%) than the left (39.0%). However, affectation of both groins constituted only 6.5% and occurred only in the males. A higher percentage of females (80.0%) had the right groin affected when compared to the males (50.8%) (Table 4). A total of 16 patients (20.8%) had a family history of hernia, with only one female having this (Table 5).

**Table 4:** Groin side affected by hernia

Side affected	Male (%)	Female (%)	Total (%)
Right	34(50.8%)	8(80.0)	42(54.5)
Left	28(41.8)	2(20.0)	30(39.0)
Both	5(7.5)	0(0.0)	5(6.5)

**Table 5:** Distribution of family history of hernia

Family history of hernia	Male (%)	Females (%)	Total (%)
Yes	15(22.4)	1(10.0)	16(20.8)
No	48(71.6)	9(90.0)	57(74.0)
Not recorded	4(6.0)	0(0.0)	4(5.2)

Duration of hospital stay ranged between two and nineteen days with median duration of six days. There were three patients that had post-operative complications. One of them had scrotal haematoma and stayed 14 days in the hospital while another one had scrotal haematoma with wound sepsis and stayed for 19 days. The third patient had wound sepsis. All the complications were successfully managed with prompt and adequate drainage and antibiotics treatment. There was no case of mesh removal due to wound infection.

## 4. Discussion

Inguinal hernias are among the commonest surgical problems, and surgical treatment of inguinal hernias constitute a significant proportion of the work load in rural general surgical practice (Ngowe et al., 2005; Pavlidis et al., 2010). In this study the proportion of males (87.0%) was greater than that of females (13.0%), while the ratio of male to females was 6.7: 1. This ratio is lower than the male to female ratio of 14:1 found by Arowolo et al. (2011) in a study in Ile Ife, Nigeria. The high female proportion in this study could be attributed to the fact that women in our environment and rural areas do perform as much strenuous work as men. Another contributory factor for this difference could be the small volume of the sample size studied.

The mean age of the patients was 46.8 ± 15.7 years even though there was no significant statistical difference in the ages of both sexes in this study. This is the mean age at which majority of the rural people engage in strenuous and laborious work. This finding is similar to the mean age of 48.78 ± 14.41 years in the study by Khan et al. (2008) and 47.2 ± 15.5 years reported by Arowolo et al. (2011).

The large proportion of farmers (25.6%) and traders (24.4%) in the study sample could be a reflection of the paucity of civil service work and the predominance of agricultural activities in the rural areas.

The duration of symptoms at presentation ranged from one year to more than two years with majority presenting between one and two years. This is a reflection of the poor attitude of rural people to their health problems. This could also be attributed to the inherent financial constraints of most rural people and ignorance to matters regarding to their health conditions.

Majority of the patients (54.5%) had their inguinal hernias on the right side while 6.5% had bilateral inguinal hernias. This finding was similar to that found by Khan et al. (2008), where 53.6% of their patients had right sided inguinal hernias while 5.4% had bilateral inguinal hernias. Inguinal hernias had been found to be more common on the right sides because of the developmental process involved in the descent of the right testis into the right scrotum.

There was family history of hernia in 20.8% of the patients. This could be attributed to the fact that when family occupation is strenuous agricultural activities, there would likely be more than one family member developing inguinal hernias. Also, hereditary inguinal anatomical abnormalities could be contributory to this observation.

Most of the procedures in this study took forty-five minutes to one hour. However a few giant hernias took longer than one hour. Generally the time reduced as more experience was gained. This procedure duration was similar to the twenty to ninety minutes duration reported by Wilson, Deans and Brough (1995) in their prospective study.

The mean duration of hospital stay of 6 days observed in this study was similar to the 5.23 days reported by Gerbitz, Rose and Eberie (2001). However, these differed from the 3 days reported by Ngowe et al. (2005) and the 2 days recorded by Wilson et al. (1995). The longer mean hospital stay in this study is a reflection of the fact that most people in our environment would prefer to stay in the hospital till non-absorbable skin sutures are removed, even when offered the option of early discharge. This might be due to the fact that the extended period of stay does not attract higher hospital bills in most private hospitals where cost of surgical treatment are pre-determined and fixed.

It was only early complications that were observed in this study since the follow-up period was short. These were only 3- scrotal haematoma, haematoma complicated by wound sepsis and wound sepsis. This gave scrotal haematoma rate of 2.5% and infection rate of 2.5% too. These low early complication rates could be attributed to the aseptic procedure, prophylactic antibiotics used and ligation of all bleeding vessels at operation fields. These findings are similar to the haematoma rate of 1.28% and infection rate of 3.84% observed by Awan, Arain, Gulzar and Yourrus (2010). Also the postoperative haematoma and wound infection complications rate of 3.3% each documented by Mahmood, Imiran and Shah (2006) are similar to the ones observed in this study.

Outcome analysis in hernia surgery is usually performed by assessing postoperative recurrence rates and long-term pain. Recurrence rate remains the most traditional outcome measure of the efficacy of hernia repair (O’Riordan & Kingsnorth, 1998). The reliable recurrence rate of inguinal hernioplasty needs over 5 years of follow-up (Paajanen & Varjo, 2010). Long-term follow-up of the patients in the environment of this study is hampered by high default rate, particularly when they are recovering satisfactorily. Challenges of telecommunication also made long term follow-up difficult, at least at the time of the study. These are limitations of this study.

## 5. Conclusion

Mesh hernioplasty is still relatively novel in our environment. Early post-operative results suggest that mesh hernioplasty could safely be done even in rural communities of West Africa, given the availability of mesh, basic medical infrastructure and relevant skilled manpower. Long term results would require studies with longer follow-up of patients. This might involve the greater use of telecommunication facilities now available in most rural communities.
